# *Pseudomonas aeruginosa* is capable of natural transformation in biofilms

**DOI:** 10.1099/mic.0.000956

**Published:** 2020-08-04

**Authors:** Laura M. Nolan, Lynne Turnbull, Marilyn Katrib, Sarah R. Osvath, Davide Losa, James J. Lazenby, Cynthia B. Whitchurch

**Affiliations:** ^1^​ The ithree institute, University of Technology Sydney, Ultimo, New South Wales, 2007, Australia; ^2^​ National Heart and Lung Institute, Imperial College London, London, SW3 6LR, UK; ^3^​ Microbes in the Food Chain Programme, Quadram Institute Bioscience, Norwich Research Park, Norwich, NR4 7UQ, UK; ^4^​ School of Biological Sciences, University of East Anglia, Norwich, NR4 7TJ, UK; ^†^​ Present address: Department of Cellular, Computational and Integrative Biology (CIBIO), University of Trento, Trento, TN 38123, Italy

**Keywords:** extracellular DNA, eDNA, genomic DNA, gDNA, horizontal gene transfer, HGT, type IV pili, T4P

## Abstract

Natural transformation is a mechanism that enables competent bacteria to acquire naked, exogenous DNA from the environment. It is a key process that facilitates the dissemination of antibiotic resistance and virulence determinants throughout bacterial populations. *Pseudomonas aeruginosa* is an opportunistic Gram-negative pathogen that produces large quantities of extracellular DNA (eDNA) that is required for biofilm formation. *P. aeruginosa* has a remarkable level of genome plasticity and diversity that suggests a high degree of horizontal gene transfer and recombination but is thought to be incapable of natural transformation. Here we show that *P. aeruginosa* possesses homologues of all proteins known to be involved in natural transformation in other bacterial species. We found that *P. aeruginosa* in biofilms is competent for natural transformation of both genomic and plasmid DNA. Furthermore, we demonstrate that type-IV pili (T4P) facilitate but are not absolutely essential for natural transformation in *P. aeruginosa *.

## Introduction

The continued increase in antimicrobial resistance (AMR) levels is considered to be a significant global threat [1]. Horizontal gene transfer (HGT) is a key source of bacterial genome variation and evolution and is largely responsible for the acquisition of antibiotic resistance genes by bacterial pathogens [2]. Bacteria can acquire and heritably incorporate new genetic information via three HGT mechanisms: conjugation, transduction and natural transformation. Conjugation is a cell-contact-dependent mechanism that transfers DNA directly from the cytoplasm of one bacterial cell into another. Transduction involves encapsidation of DNA into a bacteriophage, which then injects the DNA into the recipient cell. The third HGT mechanism is natural transformation, which involves the import of naked DNA from the environment through a specialized DNA transport apparatus [3, 4]. This is distinct from artificial transformation techniques involving electroporation and heat-shock treatments with chemically modified (e.g. CaCl_2_ and MgCl_2_) cells that are routinely used for molecular biology applications [5].

Extracellular DNA (eDNA) is present in significant quantities in both clinical and environmental settings, and provides a vast reservoir of genetic material that can be sampled by bacteria that are competent for natural transformation [6]. In many naturally competent bacterial species type-IV pili (T4P) are required for natural transformation [4]. While the exact role of T4P in natural transformation is unclear, the generally accepted model is that DNA binds to the pilus structure, which retracts and pulls the DNA to the cell surface. It is unclear whether or not DNA is translocated across the outer membrane through the PilQ secretin pore. The incoming DNA can then be accessed by the ComEA DNA translocation machinery in the periplasm, which mediates DNA uptake possibly by a ratchet mechanism [4, 7]. In Gram-positive bacterial species that do not produce T4P, natural transformation involves a number of proteins with homology to T4P proteins, which are thought to form a pseudopilus structure that spans the cell wall and is coupled to the DNA translocation complex at the cytoplasmic membrane [4, 8]. Once exogenous DNA has been taken up by the cell it can be stably incorporated into the genome via recombination or transposition, or be maintained as an independent replicon if plasmid DNA is taken up by an appropriate host [8]. Natural plasmid transformation has also been described in *Escherichia coli* [9]. This appears to be a distinct mechanism, which does not require homologues of the T4P/competence pseudopilus or DNA translocation machinery [10].

*Pseudomonas aeruginosa* is a highly antibiotic-resistant Gram-negative bacterium, which is a part of the ‘ESKAPE’ group of pathogens that pose a serious health risk worldwide. *P. aeruginosa* readily acquires antibiotic-resistance determinants, and demonstrates a high degree of genomic diversity and malleability similar to that seen in naturally transformable bacteria [11, 12]. Despite this, *P. aeruginosa* has long been thought to be incapable of natural transformation [13]. *P. aeruginosa* is a model organism for studying T4P [14]. Interestingly, *P. aeruginosa* produces copious quantities of eDNA under conditions that promote T4P production such as in static broth cultures [15], biofilms [16] and during twitching motility-mediated biofilm expansion [17, 18]. We therefore hypothesized that *P. aeruginosa* may be competent for natural transformation under conditions that promote both T4P expression and eDNA production. Here we show that some clinical and laboratory strains of *P. aeruginosa* are in fact capable of natural transformation under these conditions.

## Methods

### Strains, plasmids and growth conditions

*P. aeruginosa* strains used in this study were PAO1 (ATCC 15692 [19]), PAK [20], PA14 [21], PA103 [22], PAO1_GFP_, which contains mini-Tn7-Gm^R^-P_A1/04/03_-*egfp* encoding *gfp* and *aac1* (Gm^R^) inserted downstream of *glmS* [23], PAO1_CTX_, which contains miniCTX2 encoding *tet* (Tc^R^) inserted into the *attB* site of the chromosome [24] and T4P mutants PAK*pilA::TcR* [25], and Tn*5*-B21 mutants of *pilQ* [26], *pilT* [27]*, pilV* [28]*, fimV* [29]. The *P. aeruginosa* CF sputum clinical isolates were obtained from David Armstrong at Monash Medical Centre (Melbourne, Australia), and the otitis externa *P. aeruginosa* clinical isolates were obtained from Di Olden at Gribbles Pathology (Melbourne, Australia). The pUCPSK plasmid used is a non-conjugative *E. coli–P. aeruginosa* shuttle vector encoding *bla,* which confers carbenicillin resistance (Carb^R^) in *P. aeruginosa* [30]. *E. coli* Dh5ɑ (*recA, endA1, gyrA96, hsdR17, thi-1, supE44, relA1, φ80, dlacZΔM15*) was used as a host strain for pUCPSK and was miniprepped from *P. aeruginosa* and *E. coli* strains using a Qiagen miniprep kit according to the manufacturer’s instructions.

*P. aeruginosa* was cultured on lysogeny broth (LB) solidified with agar at 1.5 % (w/v) for routine maintenance and at 1.5 % or 1 % (w/v) for colony biofilm assays and grown in cation-adjusted Mueller–Hinton Broth (CAMHB) at 37 °C for all static broth and flow biofilm assays. Antibiotics were used at the following concentrations (w/v) as required: ampicillin 50 µg ml^−1^ for *E. coli* and carbenicillin 250 µg ml^−1^, gentamicin 100 µg ml^−1^ and tetracycline 100 µg ml^−1^ for *P. aeruginosa *.

### Bioinformatics and data and statistical analyses

Homologues of proteins involved in natural transformation were identified in *P. aeruginosa* PAO1 using blastp [31]. The Pseudomonas.com resource [32] and the PAK genome [33] were used to identify *P. aeruginosa* orthologues.

Data was graphed and analysed using Graph Pad Prism version 8.0. The number of replicates and any statistical tests are described in figure legends.

### Colony-biofilm assay

Overnight cultures of *P. aeruginosa* were grown in 2 ml CAMHB at 37 °C, shaking at 250 r.p.m. A 10 µl plastic loop was used to generate a 1 cm patch of the overnight culture (OD_600_ ≈ 6) on a dry 1 % LBA plate. This was then incubated overnight at 37 °C. The next day 10 µl of pUCPSK plasmid DNA (at a final concentration of 5 µg) was spotted onto the established colony biofilm and allowed to dry into the cells. The plate was then incubated with the agar downwards at 37 °C for the indicated time. After incubation the colony biofilm was harvested from the plate into 1 ml LB, vortexed to resuspend and then incubated at 37 °C for 30 min to fully resuspend the cells. The cell suspension was then spread plated between two 150 mm LBA plates with appropriate antibiotic selection and incubated for 24 h at 37 °C. The transformation frequency was calculated from the number of transformants/total number of harvested c.f.u.

### Static broth assay

Overnight cultures of *P. aeruginosa* were grown in 2 ml CAMHB at 37 °C, shaking at 250 r.p.m. Then,40 µl of overnight culture (OD_600_ ≈ 6) was subcultured into 2 ml fresh CAMHB with DNA added at the indicated concentration. The media, cells and DNA were then mixed and incubated at 37 °C statically for 24 h. Note for the shaking broth assay the same setup was used however the culture was incubated with shaking at 250 r.p.m. In both cases after incubation the cell suspension was then spread plated between two 150 mm LBA plates with appropriate antibiotic selection and incubated for 24 h at 37 °C. The transformation frequency was calculated from the number of transformants/total number of harvested c.f.u. To image the aggregates present in static broth cultures *P. aeruginosa* was grown as described for the static broth assay in a glass-bottomed Ibidi μ-Dish and visualized using DeltaVision Elite inverted research microscope with a ×100 10.4 numerical aperture UPlanFLN objective, InsightSSI illumination, SoftWorX acquisition software, fitted with a WeatherStation environmental chamber (Applied Precision, GE Healthcare, Issaquah, WA, USA) and a scientific CMOS 15-bit camera (pco.edge, PCO AG, Kelheim, Germany).

### Isolation of DNA for use in continuous-flow biofilm assays

Chromosomal DNA (gDNA) was purified from PAO1_GFP_ cells using the Epicentre Masterpure DNA purification kit. Extracellular DNA (eDNA) was purified from a confluent lawn of PAO1_GFP_ cultured overnight on MacConkey agar containing 5 % (v/v) glycerol. Bacteria were suspended in sterile PBS, centrifuged and the supernatant filtered through 0.2 µm PES membrane. eDNA present in the supernatant was further purified by removal of proteins and ethanol precipitation as reported previously [34]. Sterility of all DNA samples was confirmed prior to use by plating onto LB agar.

### Continuous-flow biofilm assays

In total, 10 cm lengths of Tygon laboratory tubing (2 mm ID) were inoculated with 1/100 dilution of an overnight culture (OD_600_ ≈ 6) of *P. aeruginosa* in CAMHB and allowed to attach for 2 h under static conditions after which continuous flow was commenced at a rate of 80 µl min^−1^ at room temperature. Influent media was CAMHB containing either no added DNA or DNA added at a final concentration of 1 µg ml^−1^ for pUCPSK plasmid DNA or 0.1 mg ml^−1^ for gDNA or eDNA. At harvest, the attached biofilm was removed by sonication and biofilm-associated bacteria collected by centrifugation. Transformants were selected by plating onto LB agar with appropriate antibiotic selection and incubated for 24 h at 37 °C. The transformation frequency was calculated from the number of transformants/total number of harvested c.f.u. To visualize natural transformation by PAO1 continuous-flow biofilms, an IBIDI μ-slide I (with flow kit) was inoculated and cultured as described for the Tygon tubing biofilms. Biofilms were imaged using an Olympus IX71 inverted research microscope with a ×100 10.4 numerical aperture UPlanFLN objective, FViewII monochromatic camera and AnalySIS Research acquisition software (Olympus Australia, Notting Hill, VIC, Australia) fitted with an environmental chamber (Solent Scientific, Segensworth, UK) for fluorescent imaging.

### Confirmation of natural transformation events from continuous-flow biofilms

The presence of mini-Tn7-Gm^R^-P_A1/04/03_-*egfp* at the chromosomal *attTn7* site was confirmed by PCR using primers Tn7_-up_ (5′CGTATTCTTCGTCGGCGTGAC3′) and Tn7_-down_ (5′CGAAGCCGCCGACAAGGG3′). Expression of GFP was confirmed by epifluorescence microscopy on an Olympus IX71. The presence of mini-CTX2 at the chromosomal *attB* site was confirmed by PCR using primers P_ser-up_ (5′CGAGTGGTTTAAGGCAACGGTCTTGA3′) and P_ser-down_ (5′AGTTCGGCCTGGTGGAACAACTCG 3′) [24]. To confirm the presence of pUCPSK in *P. aeruginosa *, plasmid DNA was extracted from *P. aeruginosa *, transformed into *E. coli *, extracted and confirmed by sequencing with M13_-FUP_ (5′TGTAAAACGACGGCCAGT3′).

## Results

### Natural transformation occurs within *P. aeruginosa* continuous-flow biofilms

Bioinformatic analyses of the sequenced *P. aeruginosa* strains PAO1, PA14 and PAK show that each of these strains encode homologues of all genes known to be involved in natural transformation in other bacterial species (Table 1). To determine if *P. aeruginosa* might be capable of natural transformation in biofilms, we established biofilms with a 1 : 1 mixture of PAO1_GFP_ (Gm^R^) and PAO1 [pUCPSK] (Carb^R^) under continuous-flow conditions (Fig. S1a, available in the online version of this article). PAO1_GFP_ contains mini-Tn7-Gm^R^-P_A1/04/03_-*egfp* encoding *gfp* and *aac1* (Gm^R^) inserted downstream of *glmS*.^23^ This strain was selected as PAO1 lacks a prophage capable of transduction [19] and the plasmid pUCPSK was selected as it is a non-conjugative plasmid [30], thus any observed HGT would be most likely occurring via natural transformation. Biofilm effluent was collected each day for 4 days and bacteria tested for their ability to grow on LB agar plates containing both gentamicin and carbenicillin (Fig. S1a). An average of 50–100 Gm^R^/Carb^R^ colonies (resistant to both gentamicin and carbenicillin) were obtained from the effluent of mixed PAO1_GFP_ and PAO1 [pUCPSK] biofilms on day 1 and confluent lawns of Gm^R^/Carb^R^ colonies obtained from day 2 onwards. The presence of mini-Tn7-Gm^R^-P_A1/04/03_-*egfp* at the chromosomal *attTn7* site in these colonies was confirmed by PCR amplification. To confirm that these colonies also possessed pUCPSK, plasmid DNA was extracted, transformed into *E. coli* and confirmed by sequencing. Neither the PAO1_GFP_ or PAO1 [pUCPSK] strains used to establish these mixed biofilms, or effluent from control single-strain biofilms were able to grow on the dual antibiotic selection plates. These results suggest that HGT of plasmid DNA and/or chromosomal DNA is mostly likely occurring via natural transformation in *P. aeruginosa* biofilms.

**Table 1.** Homologs of proteins involved in natural transformation in a range of bacteria

Competence protein	* Bacillus subtilis *	* Streptococcus pneumoniae *	* Haemophilus influenzae *	* Thermus thermophilus *	* Pseudomonas stutzeri *	* Neisseria gonorrhoeae *	Pseudomonas aeruginosa*
**T4P/Competence Pseudopilus**							
Traffic NTPase(s)	ComGA	ComGA	PilB	PilF	PilT, PilU	PilF, PilT	PilB, PilT, PilU
Polytopic membrane protein	ComGB	ComGB	PilC	PilC	PilC	PilG	PilC
Pilins or pseudopilins	ComGC, -GD, -GE, -GG	CglC, CglD	PilA	PilA1, -A2, -A3, -A4	PilA1	PilE, ComP	PilA, -V, -W, -X, -E, FimT, FimU
Prepilin peptidase	ComC	CilC	PilD	PilD		PilD	PilD
Secretin/pilot	na	na	ComE	PilQ		PilQ/PilP	PilQ/PilP
**DNA translocation machinery**							
DNA receptor	ComEA	ComEA		ComEA		ComE	PA3140
Membrane channel	ComEC	ComEC	Rec-2	ComEC	ComA	ComA	PA2984
ATP-binding protein	ComFA	ComFA			ExbB		PA2983
**Other**							
			DprA (Smf)				PA0021
			TfoX (Sxy)				PA4703
			CRP				Vfr
			CyaA				CyaA, CyaB
			ComM				PA5290
			ComF				PA0489

*Using *P. aeruginosa* PAO1 gene nomenclature.

To determine if HGT of chromosomal DNA did indeed occur via natural transformation, and the above result was not just due to HGT of plasmid DNA, we established biofilms with a 1 : 1 mixture of PAO1_GFP_ (Gm^R^) and PAO1_CTX_ (Tc^R^) under continuous-flow conditions (Fig. S1b). The PAO1_GFP_ used here has been described above and PAO1_CTX_ contains miniCTX2 encoding *tet* (Tc^R^) inserted into the *attB* site of the chromosome [24]. Biofilm effluent was collected each day for 8 days and bacteria tested for their ability to grow on LB agar plates containing both gentamicin and tetracycline (Fig. S1b). Whilst no Gm^R^/Tc^R^ colonies were obtained from days 1–4, from days 5–8 an average of 3 Gm^R^/Tc^R^ colonies that were resistant to both antibiotics and expressed Gfp were recovered per day. The presence of both mini-Tn7-Gm^R^-P_A1/04/03_-*egfp* and mini-CTX2 in these colonies was confirmed by PCR. Importantly, neither the PAO1_GFP_ or PAO1_CTX_ strains used to inoculate the mixed biofilms or effluent from control single-strain biofilms were able to grow on the dual antibiotic selection plates. As neither conjugation or transduction is likely to account for these HGT events in PAO1 biofilms, these results suggest that *P. aeruginosa* is able to acquire and incorporate chromosomal DNA and plasmids encoding antibiotic-resistance genes via natural transformation in biofilms.

**Fig. 1.**
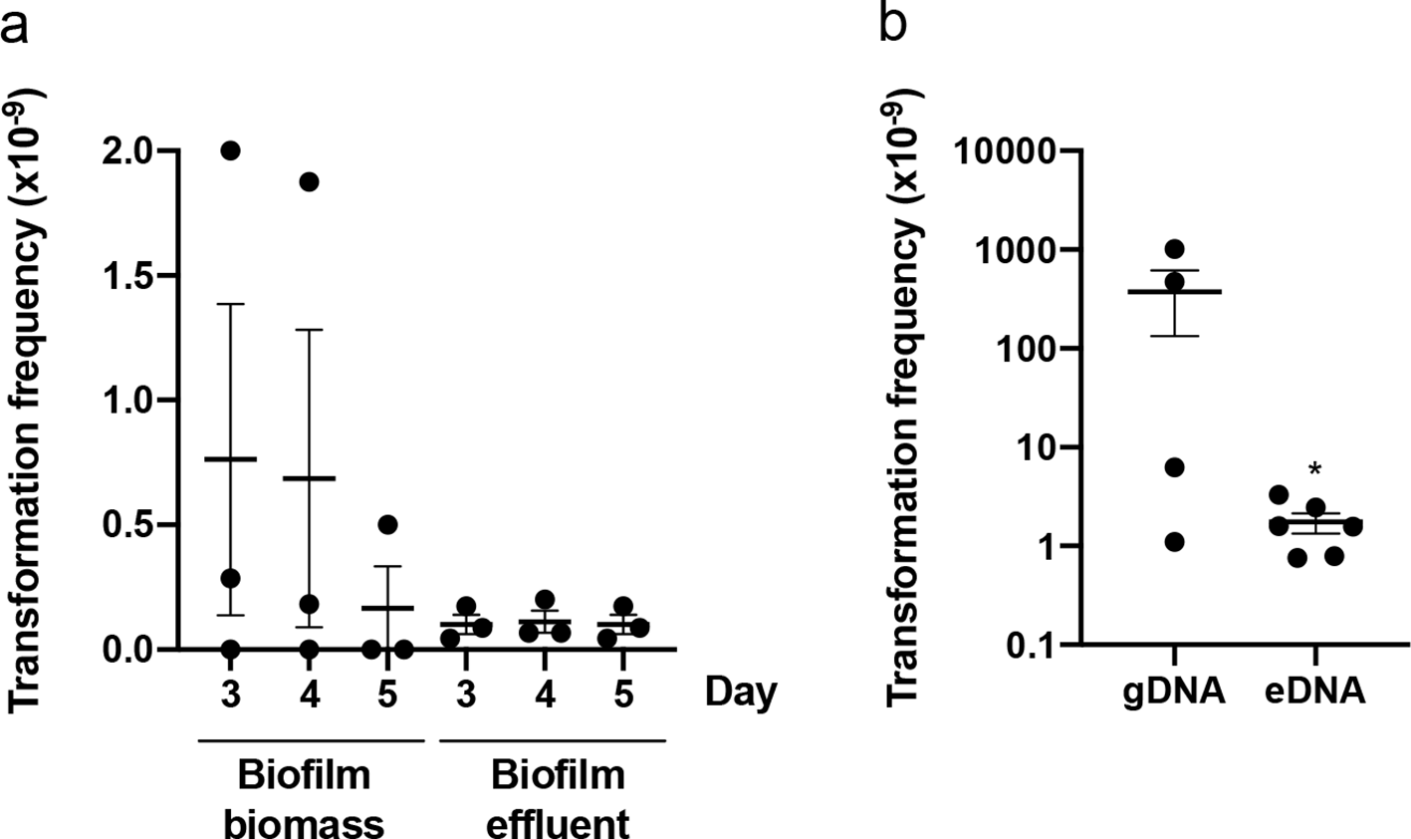
*
P. aeruginosa* is capable of natural transformation in continuous-flow biofilms. Carbenicillin- or gentamicin-resistant transformants obtained from continuous-flow biofilms of PAO1 in Tygon tubing. For (a) pUCPSK (Carb^R^) was added to the media influent (at a final concentration of 1 µg ml^−1^ with flow rate of 80 µg min^−1^) with either the biofilm biomass or effluent harvested and plated on carbenicillin selective agar plates on the indicated day. For (b) sterile gDNA or total eDNA obtained from PAO1_GFP_ (Gm^R^) (at a final concentration of 0.1 mg ml^−1^ with flow rate of 80 µg min^−1^) was added to the media influent and the biofilm biomass harvested and plated on gentamicin selective agar plates after 5 days. The mean of each set of technical triplicates was calculated to give an *n*≥4, which is presented as mean±sem (* *P* <0.05; Mann–Whitney *U*-test compared to gDNA).

To confirm that *P. aeruginosa* is indeed capable of natural transformation and to rule out any possibility of HGT through transduction or conjugation, we performed a series of experiments to follow the uptake of purified, sterile exogenous DNA. We firstly examined plasmid DNA uptake under continuous-flow biofilm conditions (Fig. S2a). PAO1-flow biofilms were cultured in the presence or absence of purified pUCPSK (Carb^R^) plasmid DNA (at a final concentration of 1 µg ml^−1^) and the amount of natural transformation within the biofilm biomass and effluent assessed at days 3, 4 and 5. Carb^R^ colonies were obtained from both the biofilm biomass and biofilm effluent (Fig. 1a). No Carb^R^ colonies were obtained in the no DNA control.

**Fig. 2.**
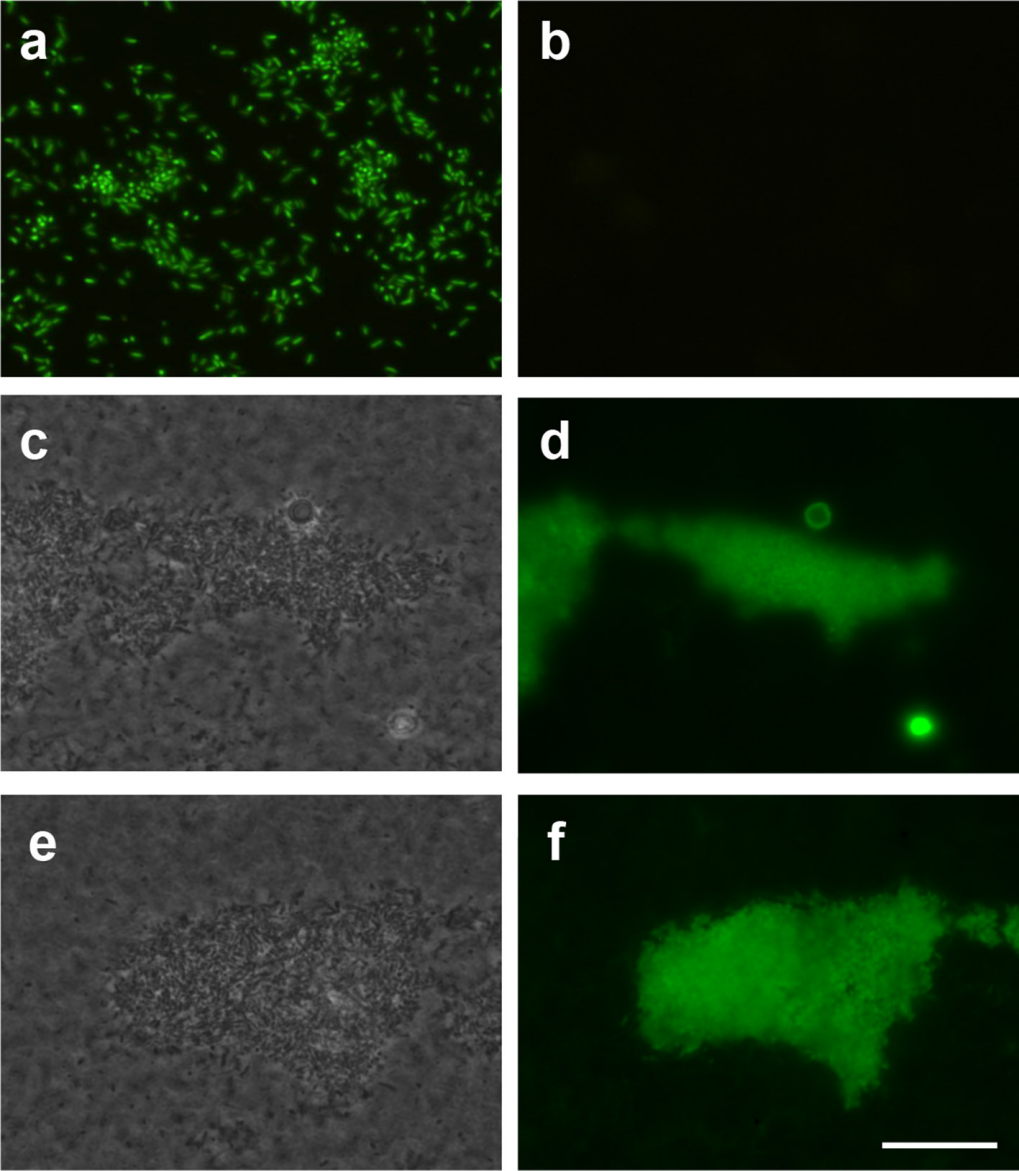
*
P. aeruginosa* cultured in continuous-flow biofilms can stably integrate and express antibiotic resistance and *gfp* gene cassettes by natural transformation of exogenous chromosomal DNA. Sterile PAO1_GFP_ gDNA (at a final concentration of 0.1 mg ml^−1^ with flow rate of 80 µg min^−1^) was added to the media influent of continuous-flow biofilms of PAO1 in (a) Tygon tubing or (c–f) flow cells and incubated for 5 days. (b) The inoculum PAO1 strain did not express GFP. (a) Gentamicin resistant (Gm^R^) colonies obtained from Tygon tubing biofilms were resuspended in PBS and visualized by epifluorescence microscopy, which showed all cells from Gm^R^ colonies expressed GFP. (c–f) The biofilm biomass from PAO1 flow cells was visualized by phase contrast (c, e) or epifluorescence microscopy (d, f), which showed the presence of representative biofilm microcolonies expressing GFP. Scale bar 100 µm.

We also examined if natural transformation by uptake of exogenous chromosomal DNA occurs in biofilms cultured under continuous flow. *P. aeruginosa* PAO1-flow biofilms were cultured in the presence and absence of sterile genomic DNA (gDNA) or extracellular DNA (eDNA) obtained from PAO1_GFP_ (Gm^R^) (at a final concentration of 0.5 mg ml^−1^) in the media influent (Fig. S2a). gDNA was purified from whole-cell lysates of PAO1_GFP_ whereas eDNA was purified from the extracellular milieu of a confluent lawn of PAO1_GFP_. After 5 days the number of Gm^R^ colonies recovered from the biofilm biomass were counted. This revealed extremely variable rates of natural transformation of gDNA by cells within the biofilm biomass across multiple experiments (Fig. 1b). This is not unexpected as the rate is likely to be dependent upon the time at which the natural transformation event occurred. If this event occurred early in the assay, we would expect many transformants recovered due to proliferation of the transformed cells. However, if transformation occurred later, we would expect far fewer transformants as these did not have as long to proliferate. For the eDNA experiments, while some Gm^R^ transformants were obtained, the rate of natural transformation was overall much lower than for gDNA (Fig. 1b). This may be due to the integrity of the DNA as we observed via agarose gel electrophoresis that the eDNA used in these experiments was quite degraded compared with the gDNA, presumably through the action of nucleases present in the extracellular milieu. No Gm^R^ colonies were obtained for continuous-flow biofilms in the absence of gDNA or eDNA indicating that the gentamicin-resistant cells recovered from these assays was due to the presence of the exogenous chromosomal DNA. To further rule out the possibility of spontaneous resistance, the presence of the mini-Tn7-Gm^R^-P_A1/04/03_-*egfp* at the chromosomal *attTn7* site in the biofilm-derived Gm^R^ colonies was confirmed by PCR. The presence of the *gfp* gene in the Gm^R^ colonies was also confirmed by visualization of GFP expression using epifluorescence microscopy (Fig. 2a). No GFP expression was observed in the PAO1 inoculum strain (Fig. 2b). As it was not possible to directly visualize biofilms cultured in Tygon tubing, PAO1 continuous-flow biofilms were cultured in transparent flow cells over 5 days in the presence and absence of gDNA obtained from PAO1_GFP_. Epifluorescence microscopy revealed microcolonies of GFP-expressing bacteria within the biofilm (Fig. 2c–f). No GFP expression was observed in the no DNA control biofilms.

### Natural transformation occurs within colony biofilms of *P. aeruginosa*

**Fig. 3.**
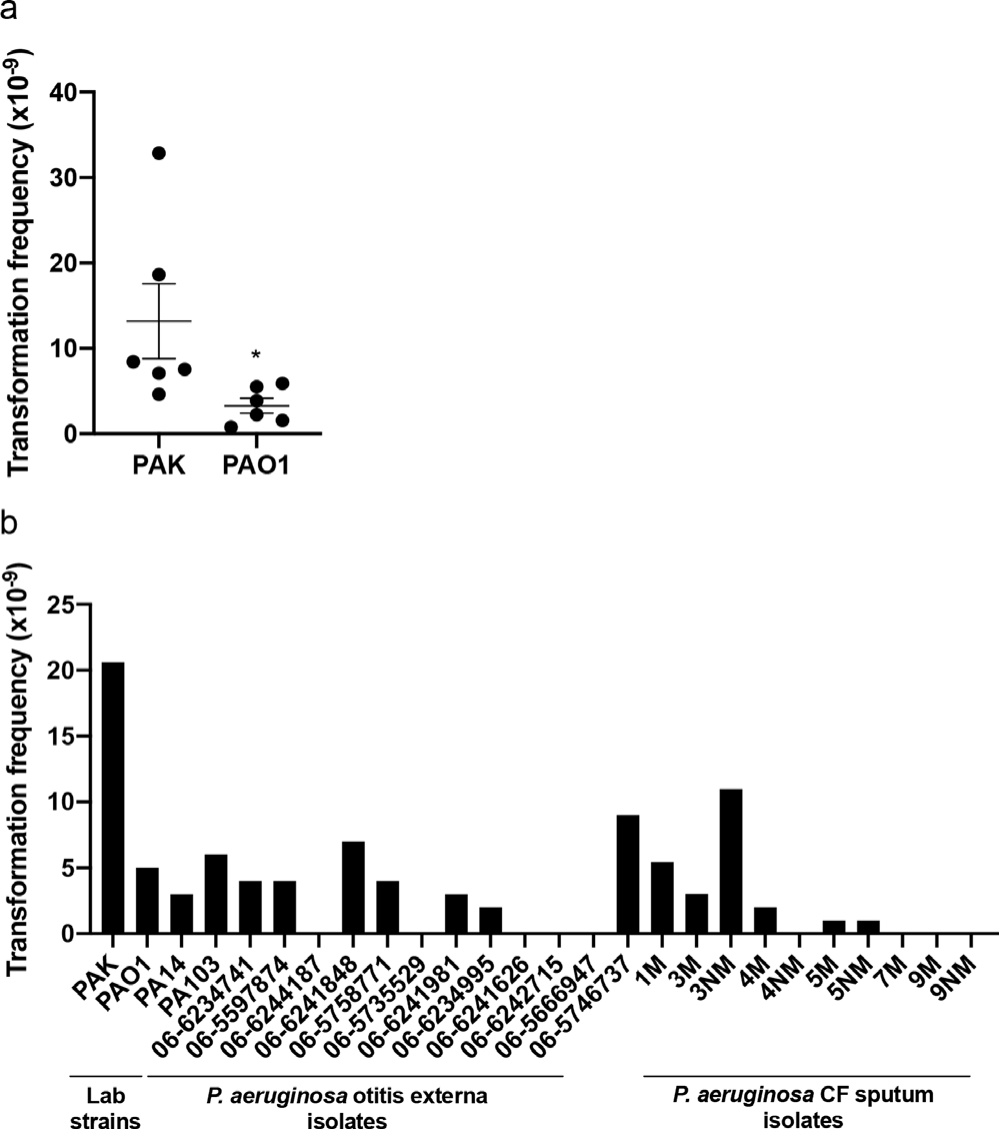
Lab and clinical strains of *P. aeruginosa* are capable of natural transformation of plasmid DNA within colony biofilms. pUCPSK (5 µg) DNA was applied to colony biofilms of (a) *P. aeruginosa* PAK or PAO1, or (b) *P. aeruginosa* lab or clinical strains and incubated at 37 °C for 2 h. Cells were then harvested and the number of carbenicillin-resistant transformants determined by spread plating on selective media. For (a) the mean of each set of technical triplicates was calculated to give an *n*=6, which is presented as mean±sem (* *P* <0.05; Mann–Whitney *U*-test compared to PAK). For (b) the values presented are from *n*=1. For the CF sputum isolates the designation M refers to mucoid phenotype, NM is non-mucoid.

To further investigate natural transformation by *P. aeruginosa* we set out to determine if this process was also occurring in colony biofilms on agar plates. We first grew wild-type strains PAK and PAO1 on LB agar overnight to form a colony biofilm on the surface of the agar. We then added 5 µg of sterile DNA (or the equivalent volume of sterile water) of the plasmid pUCPSK (Carb^R^), onto the surface of the colony biofilm. After 2 h incubation at 37 °C the colony was resuspended and cells plated onto media containing carbenicillin to select for transformants that had acquired the plasmid (Fig. S2b). Only colony biofilms that had been exposed to plasmid DNA yielded Carb^R^ colonies (Fig. 3a), whereas colony biofilms exposed to sterile water yielded none. The transformation frequency of PAK was significantly higher than that of PAO1 [13.2±4.4 and 3.3±0.7 transformants/c.f.u. (×10^−9^), respectively]. To confirm that the carbenicillin-resistant colonies had acquired pUCPSK, plasmid DNA was extracted, re-transformed into *E. coli* and confirmed by sequencing. These observations indicate that a proportion of cells within colony biofilms of *P. aeruginosa* are competent for natural transformation and are able to take up and maintain plasmid DNA.

Given that both *P. aeruginosa* strains PAK and PAO1 appeared to be naturally competent, we wanted to determine if this was also the case for two other commonly utilized lab strains (PA14 and PA103) and clinical isolates. All *P. aeruginosa* strains were first confirmed to be carbenicillin sensitive prior to use. Twelve *P. aeruginosa* otitis externa and ten cystic fibrosis (CF) lung sputum isolates were assayed for the ability to uptake pUCPSK plasmid DNA in a colony biofilm. Of these, 7/12 otitis externa and 6/10 CF isolates were able to uptake exogenous plasmid DNA (Fig. 3b). Interestingly the site of infection, or the mucoidy status did not appear to impact natural transformation ability. No Carb^R^ colonies were obtained in the no plasmid DNA controls for each strain. Interestingly, transformation ability varied amongst both clinical and lab strains. Of the lab strains, PA14 was the least capable of natural transformation with PAK the most efficient. These results demonstrate that many lab and clinical isolate strains of *P. aeruginosa* are capable of natural transformation within colony biofilms, albeit with different efficiencies.

### Natural transformation occurs within static broth cultures of *P. aeruginosa*

**Fig. 4.**
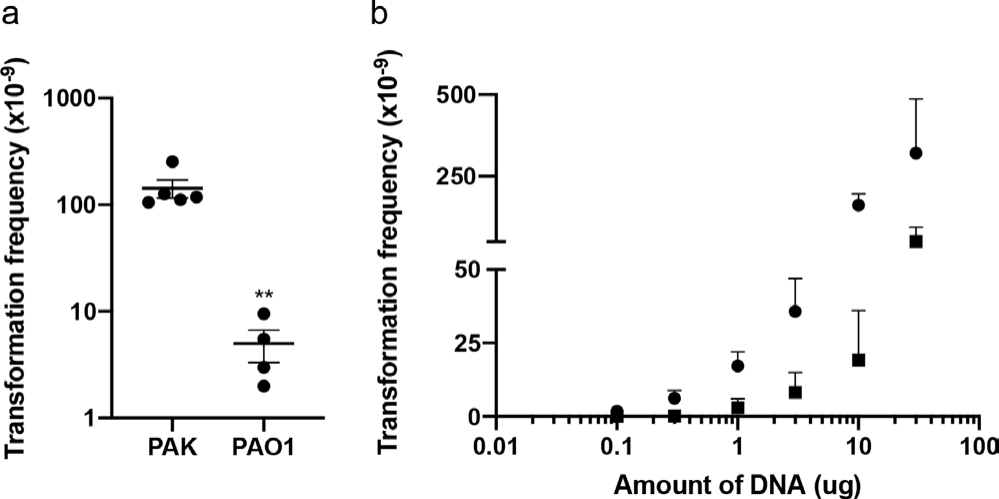
*
P. aeruginosa* is capable of natural transformation of plasmid DNA in static broth cultures. Carbenicillin-resistant transformants obtained from static broth cultures incubated at 37 °C for 24 h with (a) 10 µg pUCPSK or (b) 0.1–30 µg pUCPSK with PAK (circles) or PAO1 (squares). The mean of each set of technical triplicates was calculated to give an *n*≥3, which is presented as mean±sem. For (a) ** *P*<0.005; Mann–Whitney *U*-test compared to PAK. For (b) there was no significant difference (*P*>0.05; Friedman with Dunn’s multiple comparisons test) between DNA concentrations (0.1–30 µg) for both strains.

*P. aeruginosa* also expresses T4P when cultured in static nutrient broth [14]. Under these conditions *P. aeruginosa* forms biofilms and suspended microcolony aggregates that contain eDNA [15, 35]. To determine if natural transformation also occurred in static broth cultures, 10 µg of sterile pUCPSK plasmid DNA (or the equivalent volume of sterile water) was added to a subculture of *P. aeruginosa* wild-type PAK or PAO1 and incubated statically at 37 °C for 24 h. Cells were then recovered and plated onto media containing carbenicillin to select for transformants (Fig. S2c). Carb^R^ colonies were obtained for both PAO1 and PAK under these conditions, whereas no Carb^R^ colonies were identified in the water control. As was observed with colony-biofilm transformations (Fig. 3a), PAK was significantly more efficient for natural transformation of pUCPSK than PAO1 in static broth cultures, with efficiencies of 143.3±27.7 and 5.0±1.7 transformants/c.f.u. (x10^−9^), respectively (Fig. 4a).

To determine if transformation frequency was dependent on the amount of DNA added, we performed static broth transformation assays with increasing amounts of plasmid DNA from 0.1 to 30 µg. As expected, transformation frequency increased with increasing amounts of sterile plasmid DNA added (Fig. 4b).

We were also interested in determining whether *P. aeruginosa* was also able to uptake exogenous chromosomal DNA and integrate this into the chromosome in static broth cultures. To examine this, chromosomal DNA from PAO1_GFP_ (Gm^R^) was purified from either a sterile whole-cell lysate (gDNA) or the total sterile (cell-free) DNA from a confluent agar plate culture (eDNA) and 15 µg added to static broth cultures of PAK or PAO1 for 24 h. Cells were recovered and cultured on agar containing gentamicin to select for transformants. These assays revealed that natural transformation of gDNA occurred at a low frequency for both PAK and PAO1 in static broth cultures (Fig. S3). No natural transformation with sterile eDNA was observed in static broth cultures for either PAK or PAO1 (Fig. S3). As mentioned above for eDNA in continuous-flow biofilms, this may be due to the degraded nature of the eDNA compared to the gDNA as visualized by agarose gel electrophoresis. No gentamicin-resistant colonies were obtained in the no DNA controls.

### T4P are not absolutely required for natural transformation by *P. aeruginosa*

**Fig. 5.**
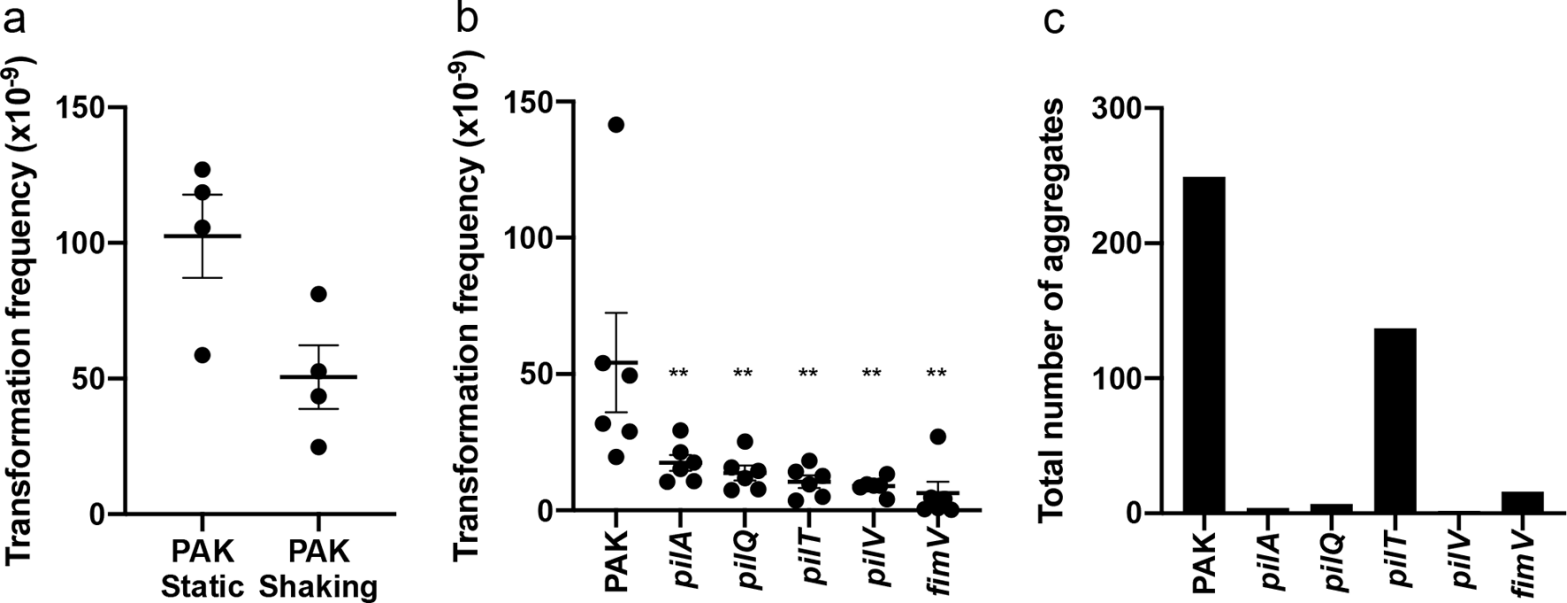
T4P are not absolutely required for natural transformation. Static (a–c) or shaking (a) broth cultures were incubated at 37 °C for 24 h with 10 µg pUCPSK and the number of (a, b) carbenicillin-resistant transformants or (c) aggregates determined. For (a, b) the mean of each set of technical triplicates was calculated to give an *n*≥3, which is presented as mean±sem. For (a) *P* >0.05 (ns); Mann–Whitney *U*-test. For (b) ** *P* <0.005; Mann-Whitney *U*-test compared to PAK. For (c) the total number of aggregates from 60 random fields imaged on 3 separate days (20 fields/day) is presented.

*P. aeruginosa* produces more T4P when cultured in static broth cultures than under shaking conditions [14]. We investigated the effects on natural transformation frequency of culturing under static or shaking conditions and found that although some natural transformation was still observed under shaking conditions, more transformants were obtained with static culture conditions (Fig. 5a), consistent with a role of T4P in natural transformation. To directly examine the role of T4P in natural transformation of *P. aeruginosa,* we added 10 µg sterile pUCPSK plasmid DNA to static broth cultures of PAK mutants defective in the production of the pilin subunit (*pilA*), in T4P assembly (*pilV*, *pilQ, fimV*) and in T4P retraction (*pilT*). Interestingly, all T4P mutants were capable of some natural transformation of pUCPSK, however a significant reduction in transformation frequency compared to wild-type PAK was observed (Fig. 5b). No Carb^R^ colonies were identified in the no DNA controls. There was no apparent difference in the transformation frequency of mutants, which either did not have any surface-assembled T4P (*pilA, pilV, pilQ, fimV*) or were unable to retract T4P (*pilT*) (Fig. 5b).

Given that wild-type *P. aeruginosa* forms aggregates in a static broth [15, 35] we considered the possibility that natural transformation was occurring within these aggregates in our static broth transformation assay. To determine if the reduction in transformation frequency by T4P mutants (Fig. 5b) could be accounted for by the inability to form aggregates, we used microscopy to image wild-type PAK and T4P mutants under static broth conditions. This revealed that mutants unable to make surface-assembled T4P (*pilA, pilV, pilQ, fimV*) were also unable to form aggregates (Fig. 5c). A *pilT* mutant, which has a T4P retraction defect, was however still able to form aggregates (Fig. 5c). While the total number of aggregates was less than that observed for wild-type (Fig. 5c), this decrease does not account for the observed decrease in natural transformation frequency (Fig. 5b). This suggests that it is the lack of functional T4P that affects natural transformation and not other factors associated with aggregate formation.

Overall these data suggest that in *P. aeruginosa *, T4P facilitate transport of DNA to the cell surface but are not essential for natural transformation, at least of plasmid DNA. Furthermore, these observations indicate that during natural transformation in *P. aeruginosa* the PilQ secretin pore is not absolutely required for translocation of plasmid DNA across the outer membrane.

## Discussion

Here we have demonstrated, in contrast to current dogma, that *P. aeruginosa* is capable of natural transformation of both plasmid and chromosomal DNA under conditions that promote the expression of T4P and eDNA production, such as in static broth cultures and biofilms. We found that whilst T4P appear to be involved in facilitating plasmid DNA uptake, T4P are not absolutely required for natural transformation of plasmid DNA in *P. aeruginosa *. This is similar to what has been reported in *Vibrio cholerae* for gDNA uptake [36]. Furthermore, our data suggests that the PilQ secretin pore is not absolutely required for translocation of plasmid DNA across the outer membrane in this organism. Given that T4P play some role in this process suggests that regions of a biofilm with higher levels of T4P expression may display increased levels of natural transformation, for example the actively expanding edge of a colony biofilm [37] and within the mushroom caps of a fully hydrated biofilm [38], though this is yet to be determined.

It is thought that separate uptake mechanisms for plasmid or chromosomal DNA are present in *E. coli* [9]. While natural transformation of chromosomal DNA and integration into the genome has not been demonstrated in *E. coli *, T4P/competence pseudopilus and DNA translocation machinery homologs have been shown to be responsible for uptake of chromosomal DNA for use as a nutrient source [39]. Natural plasmid transformation in *E. coli* does not utilize the competence pseudopilus uptake system although the precise mechanism for this process has not been determined [10]. At this stage we cannot rule out the possibility that there are two mechanisms in *P. aeruginosa* involved in the natural transformation of plasmid DNA or chromosomal DNA.

The finding that *P. aeruginosa* is capable of natural transformation is a paradigm shift in our understanding of how this pathogen acquires genetic diversity. Indeed, recombination has been identified as a major means of genetic diversity in many *P. aeruginosa* clinical isolates although the source of DNA and the mechanism of HGT was not determined [12, 40–42]. Natural transformation may be an important mechanism for the acquisition of antibiotic resistance and virulence genes in this ESKAPE pathogen and a significant contributor to the rapid increase in the number of multidrug-resistant *P. aeruginosa* strains that are an emerging problem worldwide.

## Supplementary Data

Supplementary material 1
