# Supplementary material for: Pseudomonas aeruginosa is capable of natural transformation in biofilms
Source: Microbiology (Reading). 2020 Aug 4;166(10):995–1003. doi: 10.1099/mic.0.000956 (PMC7660920; doi:10.1099/mic.0.000956)
Supplement: Supplementary material 1 [file mic-166-995-s001.pdf]

Pseudomonas aeruginosa is capable of natural transformation in biofilms

Laura M. Nolan[1, 2], Lynne Turnbull[1], Marilyn Katrib[1], Sarah R. Osvath[1], Davide Losa[1], James J. Lazenby[3] and Cynthia B. Whitchurch[1,3,4]*

Author affiliations:

[1] The ithree institute, University of Technology Sydney, Ultimo, New South Wales, 2007, Australia.

[2] National Heart and Lung Institute, Imperial College London, London, SW3 6LR, UK.

[3] Microbes in the Food Chain Programme, Quadram Institute Bioscience, Norwich Research Park, Norwich, NR4 7UQ, UK

[4] School of Biological Sciences, University of East Anglia, Norwich, NR4 7TJ, UK

* **Corresponding author:**

Email: Cynthia.Whitchurch@quadram.ac.uk

Supplementary Figures

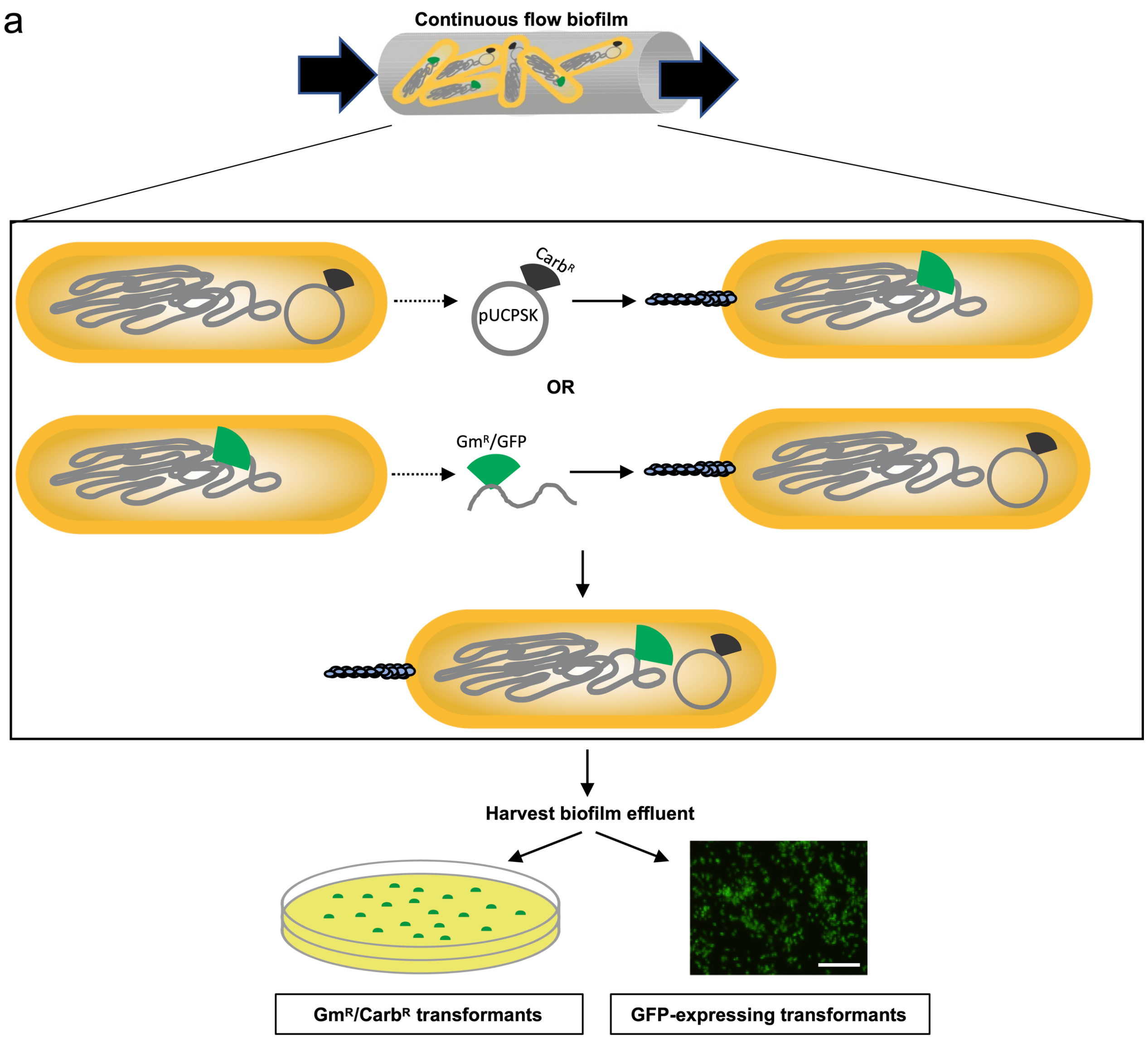

Supplementary Figure 1. Natural transformation by *P. aeruginosa* within mixed species biofilms. Continued next page

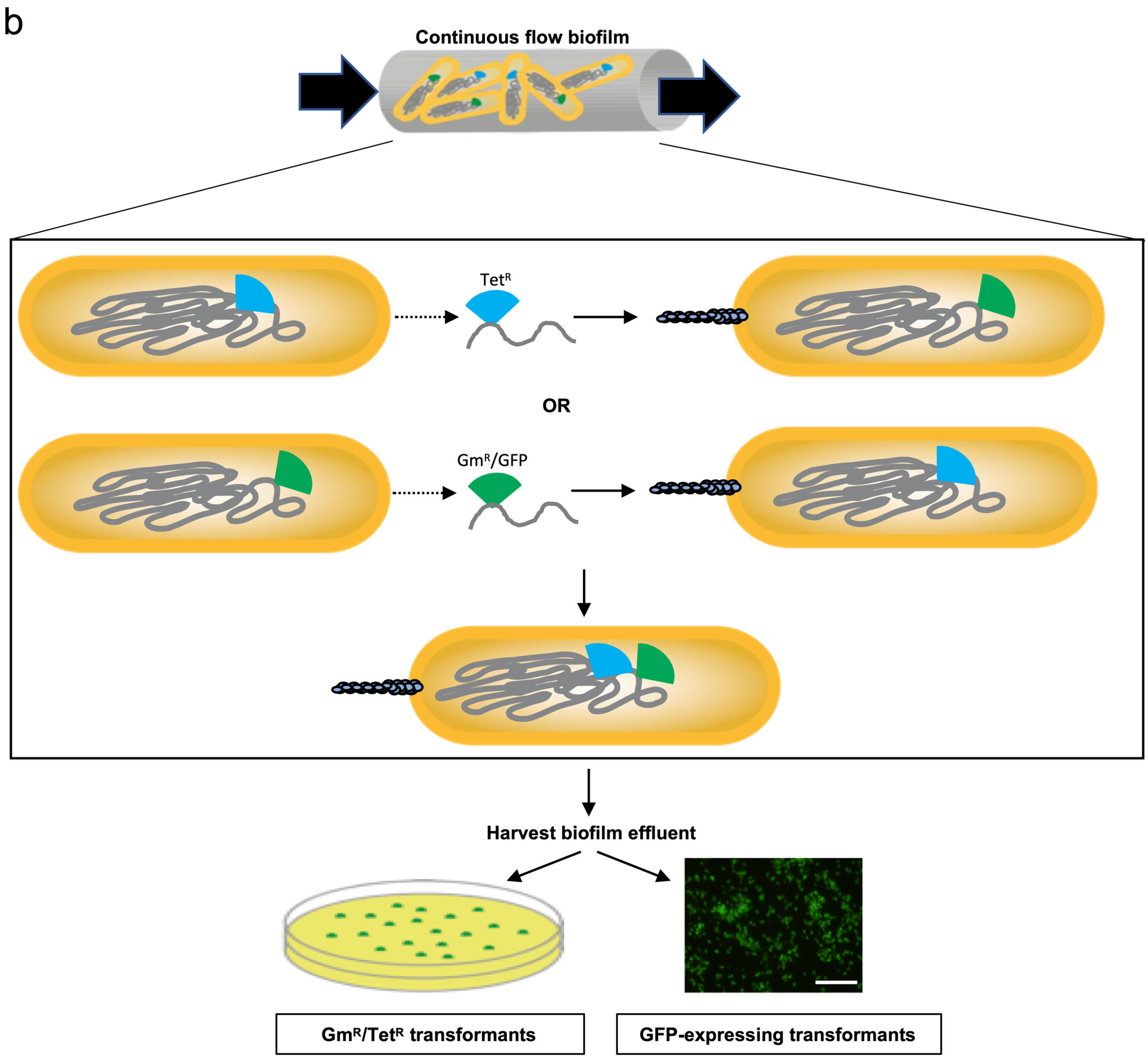

Supplementary Figure 1. Natural transformation by *P. aeruginosa* within mixed species biofilms. (a) PAO1$_{GFP}$ (Gm^{R}) and PAO1 with pUCPSK (Carb^{R}) or (b) PAO1$_{CTX}$ (Tc^{R}) and PAO1$_{GFP}$ (Gm^{R}) are capable of gDNA or plasmid DNA exchange in mixed flow biofilms. Continuous flow biofilms were established and cells allowed to exchange (a) plasmid (Carb^{R}) and/or gDNA (Gm^{R}/GFP) or (b) gDNA (Tc^{R} and/or Gm^{R}/GFP) relying to some extent upon Type-IV pili (blue appendage at cell pole) for uptake into recipient cell. The resulting transformants were collected from the biofilm effluent every 24 h and plated onto selective media (Carb^{R} and Gm^{R} or Tet^{R} and Gm^{R}) and imaged using fluorescence microscopy to observed GFP expression. Scale bar 100 µm.

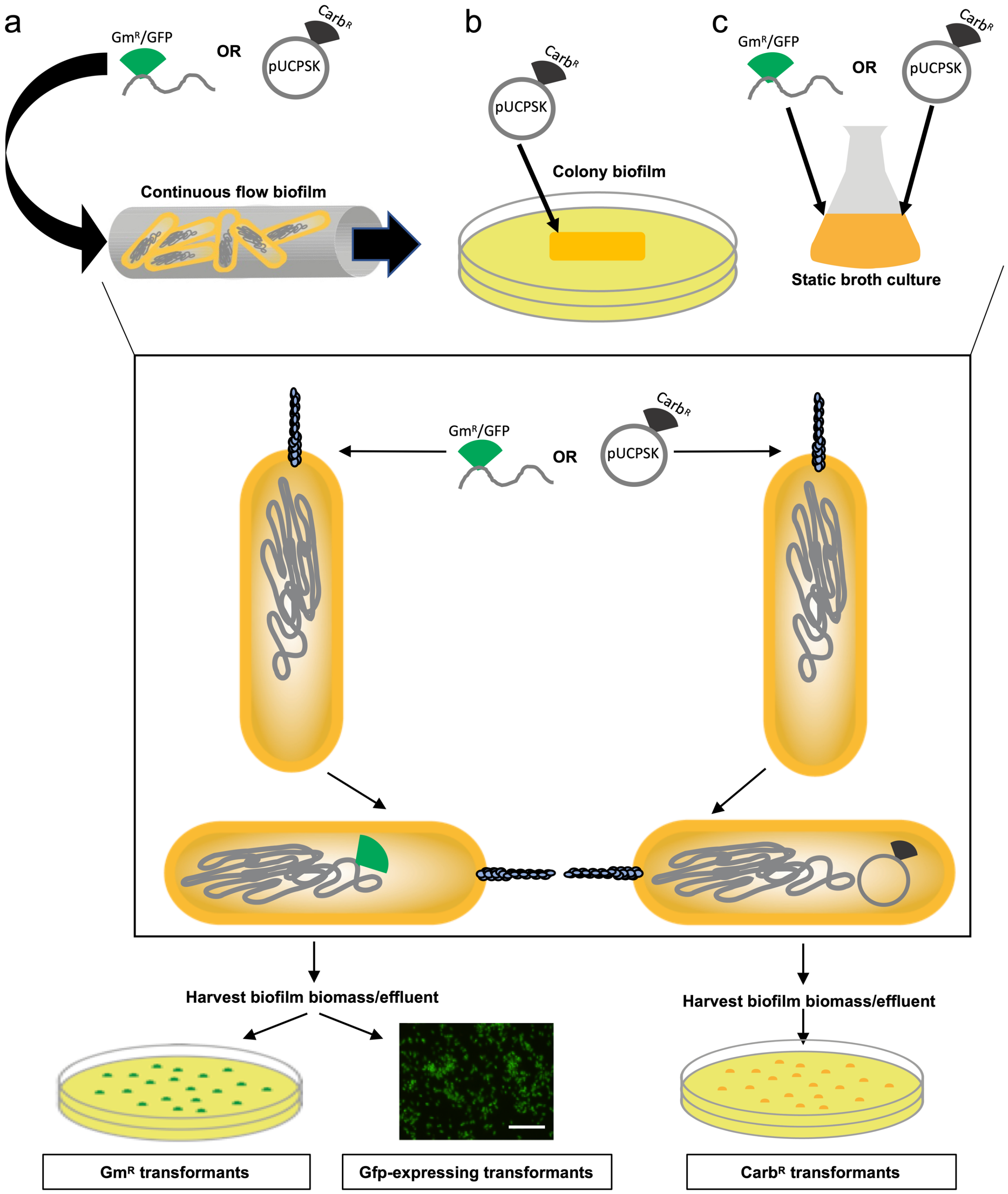

Supplementary Figure 2. Uptake of exogenous DNA via natural transformation in *P. aeruginosa* biofilms. The indicated exogenous DNA (either gDNA from PAO1$_{GFP}$ (Gm^R/GFP) or pUCPSK plasmid DNA (Carb^R)) was added to (a) the media influent of continuous flow biofilms, (b) established colony biofilms or (c) static broth cultures of *P. aeruginosa.* The ability of cells to uptake either exogenous source of DNA was dependent

to some extent upon Type-IV pili (blue appendage at cell pole). The transformation frequency was determined by harvesting biofilm biomass (and effluent for (a) or aggregates for (b)) and plating on selective plates to identify transformants that were Gm^R and expressing GFP or $Carb^R$. Scale bar 100 μm.

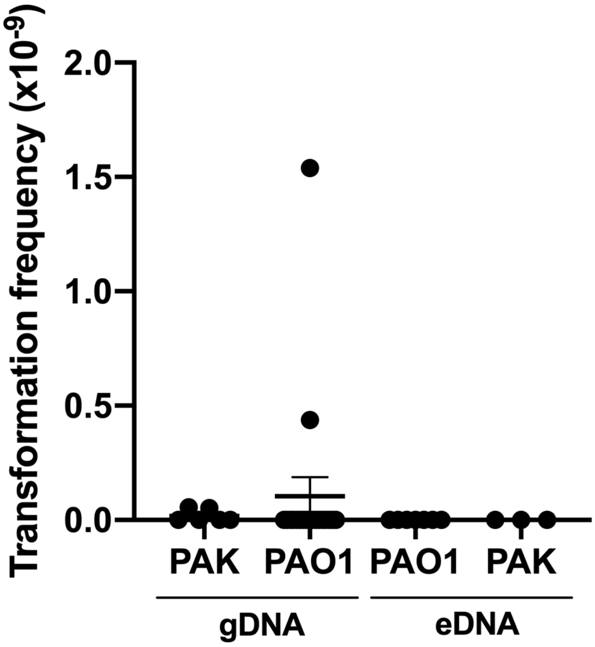

Supplementary Figure 3. Rates of natural transformation of gDNA and eDNA are low in static broth cultures of *P. aeruginosa*. Static broth cultures of PAK or PAO1 with 15 µg gDNA or eDNA from $PAO1_{GFP}$ (Gm^R) were incubated at 37°C for 24 h and the numbers of gentamicin resistant transformants determined. The mean of each set of technical triplicates was calculated to give an n≥3 which is presented as mean ± SEM.
